# Resolving the paradox of bootleg innovation: the role of organizational error management climate in shaping innovation among new-generation employees

**DOI:** 10.3389/fpsyg.2025.1646489

**Published:** 2025-12-11

**Authors:** Bingqian Zhao, Xiushen Hu, Zimeng Zhang, Yingying Guo

**Affiliations:** 1 School of Economics and Management, China University of Mining and Technology, Xuzhou, China; 2 School of Business Administration, Huaqiao University, Quanzhou, China; 3 School of Economics and Management, Guangzhou University of Applied Science and Technology, Zhaoqing, China; 4 International Business School, Hainan University, Haikou, China

**Keywords:** error management climate, psychological safety, bootleg innovation, promotionfocus, defensive focus

## Abstract

**Introduction:**

Bootleg innovation is prevalent among new-generation employees, but the influence of organizational error tolerance remains underexplored.

**Methods:**

Two studies were conducted—a three-wave field survey (*n* = 387) and a scenario-based experiment (*n* = 200)—to test the proposed model.

**Results:**

Error management climate positively predicts bootleg innovation via psychological safety. Promotion focus strengthens this mediation, while defensive focus weakens it.

**Discussion:**

The findings highlight how organizational climate and individual motivation interact to shape constructive deviant innovation.

## 1 Introduction

Organizations in today’s dynamic and resource-constrained environments are increasingly seeking to stimulate employee-driven innovation. However, this ambition often reveals a paradox: while organizations promote autonomy and creativity, they also impose formal rules and control systems that can inadvertently restrict innovative behaviors (Augsdorfer, 2012; Bennett et al., 2024). As a result, employees may bypass formal procedures and engage in unauthorized yet purposeful innovation activities, termed bootleg innovation, intended to benefit the organization (Jiang et al., 2023). Despite its informal and even subversive nature, bootleg innovation is widespread, especially in highly innovation sectors. This phenomenon presents a critical challenge for organizations: how to manage these unofficial innovation behaviors in ways that preserve their creative value without violating organizational discipline.

This challenge is particularly pronounced among new-generation employees, born in the 1990s and 2000s, who now make up a significant portion of the workforce in many organizations. These employees typically exhibit higher levels of education, open-mindedness, and a strong drive for innovation, while showing less tolerance for bureaucratic constraints (Zhou and Qian, 2021). This group’s distinct characteristics—such as a greater openness to non-traditional work practices, a strong inclination towards autonomy, and higher risk-taking tendencies—make them particularly relevant for understanding how organizational climates influence innovation behaviors. Their desire for innovation and greater autonomy make them more likely to engage in bootleg innovation, which is a crucial but underexplored aspect of organizational behavior in today’s workforce. Compared to older cohorts, they are better positioned to leverage organizational resources for creative initiatives. However, their lack of experience and desire for autonomy can lead to greater conflict with organizational norms and an increased propensity for bootleg innovation. The informal nature of such innovation, coupled with limited organizational support, also raises the likelihood of errors during the innovation process (Appelbaum et al., 2007), potentially resulting in both task-related setbacks and psychological strain. These characteristics make the new generation a compelling and timely focus for research on bootleg innovation.

Although prior studies have examined various antecedents of innovation behavior, such as personality traits (Mount et al., 2006), psychological empowerment (Zhang et al., 2025), organizational climate (Urbancová, 2013; Ghosh, 2015), job characteristics (Huang et al., 2024), and leadership styles (Podsakoff et al., 2006), little is known about how employees’ perceptions of organizational error tolerance shape their willingness to engage in bootleg innovation, particularly among new-generation employees. This group’s unique traits, such as their openness to new ideas, desire for autonomy, and limited tolerance for traditional structures, make them especially important for understanding how error management climates affect innovation behaviors. This is a critical omission because, given the trial-and-error nature of bootleg innovation, the fear of failure and negative evaluation may strongly influence employees’ risk-taking behaviors (Xu et al., 2020; Courtois and Gendron, 2017).

To address this gap, we focus on error management climate (EMC), a shared perception among employees that mistakes are tolerated, openly discussed, and viewed as opportunities for learning (Van Dyck et al., 2005). While EMC has been widely studied at the organizational level, influencing outcomes such as organizational performance (Van Dyck et al., 2005), organizational learning (Bell et al., 2009), and innovation (Bledow et al., 2009; Carless and De Paola, 2000), research at the individual level remains relatively sparse. Existing studies have shown that EMC affects individual-level outcomes such as safety performance (Maurer and Lippstreu, 2017), error reporting (Farndale and Truss, 2015), psychological empowerment (Zhang and Li, 2020), work stress (Zhang et al., 2024), and individual innovation (Chen et al., 2022). However, these studies often focus on R&D staff or senior employees, largely overlooking new-generation workers who are increasingly central to organizational innovation efforts. This omission is especially problematic given the rapid generational shifts in today’s labor force.

Thus, how new-generation employees perceive error management climate and how this shapes their engagement in bootleg innovation is an important but underexplored question. To understand this relationship, we draw on Conservation of Resources (COR) theory (Halbesleben et al., 2014), which posits that individuals seek to protect existing resources and invest in opportunities to acquire new ones. A tolerant error climate can reduce employees’ perceived risk of resource loss (e.g., damaged reputation or supervisor punishment) while fostering learning, experimentation, and resource gain. By signaling that errors will not lead to severe consequences, EMC can encourage new-generation employees to engage in innovation risk-taking behaviors like bootleg innovation (Eva et al., 2019).

We further propose that psychological safety, a belief that one will not be punished or humiliated for taking interpersonal risks (Edmondson, 1999), plays a mediating role in this relationship. EMC is likely to enhance psychological safety, which in turn facilitates innovation by reducing anxiety about making mistakes. Moreover, based on Social Information Processing Theory (Salancik and Pfeffer, 1978), we suggest that employees interpret organizational signals differently depending on their trait regulatory focus, a stable personality trait reflecting whether individuals are oriented toward growth (promotion focus) or security (defensive focus) (Higgins et al., 1997; Lanaj et al., 2012). While COR theory helps explain how EMC reduces the perceived risk of resource loss (e.g., damage to reputation or supervisor punishment), regulatory focus theory complements this by showing how individual motivations, such as a promotion focus or defensive focus, influence how employees interpret and respond to organizational error management signals. Together, these frameworks provide a more integrated view of how EMC influences bootleg innovation among new-generation employees.

This study makes three key contributions to the literature on innovation and organizational behavior. First, it expands research on bootleg innovation by examining how employees’ perceptions of error management culture rather than solely formal innovation support shape their unauthorized innovation behaviors. Second, by integrating conservation of resources theory with psychological safety, this study offers a nuanced psychological mechanism to explain why and when employees are willing to take the risk of engaging in bootleg innovation. Third, drawing on social information processing theory, we highlight the moderating role of trait regulatory focus, identifying important boundary conditions under which error management climate fosters innovation. In doing so, this research contributes to a deeper understanding of how organizations can strategically navigate the tension between compliance and creativity, particularly among younger, autonomy-seeking employees.

## 2 Literature review and hypothesis development

### 2.1 Error management climate and bootleg innovation of new-generation employees

In complex and dynamic environments, coupled with the inherent limits of human rationality, mistakes are inevitable during work. Consequently, researchers have adopted more constructive, scientifically grounded approaches to understanding and managing errors. An error management climate (EMC) refers to employees’ shared perceptions of organizational rules, values, and practices regarding mistakes (Guchait et al., 2020). While Error Management Culture often emphasizes deeper organizational values and norms, Error Management Climate focuses on observable practices and shared perceptions that employees can more directly assess. This distinction between culture and climate is important, as climate is typically associated with visible practices that reflect the immediate work environment, making it more tangible and measurable for employees’ perceptions (Van Dyck et al., 2005).

Bootleg innovation is not simply a blend of transgression and creativity; it represents a distinct form of innovation that arises when employees, faced with limited resources and constrained by organizational norms, pursue ideas that conflict with existing rules and regulations (Criscuolo et al., 2014). Although bootleg innovation violates formal norms, its underlying motivation is to advance organizational well-being. In essence, it constitutes a pro-organizational form of deviance, in which employees intentionally deviate from stated policies to achieve legitimate, higher-order organizational or moral objectives (Galperin, 2012). Drawing on Conservation of Resources (COR) theory (Hobfoll, 2011; Halbesleben et al., 2014), this paper examines how EMC influences employees’ bootleg innovation.

Resource conservation theory posits that in innovation-oriented environments, an error management climate provides employees with crucial organizational and psychological resources, thereby encouraging greater engagement in bootleg innovation (Halbesleben et al., 2014). Because this climate is fault-tolerant, employees feel protected from punishment for mistakes and experience less frustration when errors occur. Consequently, they are less likely to attribute failures to personal inadequacies (Fischer et al., 2018) and retain confidence in navigating the uncertainties and risks of innovation. These psychological resources help reduce anxiety and emotional exhaustion resulting from mistakes (Clore et al., 2014), and they mitigate negative outcomes such as guilt, pressure, and tension caused by resource depletion and wasted effort (Fan et al., 2023). Moreover, they offset the resource losses that younger employees may incur when engaging in bootleg innovation. In this way, an error management climate empowers younger employees to take risks and pursue innovative practices.

Furthermore, the resource investment principle of conservation of resources theory posits that individuals with abundant initial resources are intrinsically motivated to obtain additional resources, often investing what they already possess to create a self-reinforcing cycle of resource accumulation (Ferdous, 2016). An organizational error management climate represents a critical internal factor: it not only helps employees identify and address problems but also grants them access to supplementary learning opportunities. By encouraging employees to view mistakes with a constructive, scientific mindset, EMC enhances their perception of innovation as legitimate, reduces concerns about uncertainty, and helps overcome cognitive inertia. Even when innovation activities occur informally or privately, EMC channels these behaviors toward organizational benefit, maximizing value and facilitating further resource gains (Maurer et al., 2017). Based on these observations, this paper proposes the following hypothesis:

*H1:* An error management climate positively influences bootleg innovation among new-generation employees.

### 2.2 Mediating effect of psychological safety

Psychological safety, defined as employees’ perception that they can take interpersonal risks at work without fear of negative consequences, is strongly influenced by organizational climate (Edmondson, 1999). An error management climate (EMC) promotes inclusivity, error analysis, communication, and learning, encouraging employees to view mistakes positively and reducing fears of punishment (Frazier et al., 2017). Consequently, EMC enhances psychological safety, alleviating the anxiety and emotional exhaustion that often accompany uncertain, error-prone behaviors such as bootleg innovation. With higher psychological safety, employees feel secure enough to pursue bootleg innovation without fearing adverse repercussions.

From a resource conservation theory perspective, psychological safety represents a key supportive resource that an organization provides (Hobfoll, 2011). In secure environments, employees perceive lower uncertainty and risk, making them more willing to embrace challenging tasks. Conversely, when the environment feels threatening, heightened risk perceptions prompt preventive behaviors (Brashers, 2007). Bootleg innovation inherently involves both material and interpersonal risks; thus, a strong sense of psychological safety is essential to sustain such risky, trial-and-error efforts (Appelbaum et al., 2007). When employees succeed in bootleg innovation, they typically gain additional work-related resources, which they reinvest, creating a positive feedback loop of resource accumulation (Ferdous, 2016).

EMC fosters this supportive context by encouraging employees to share and learn from mistakes rather than conceal them, thereby reducing perceived threats and alleviating mental pressure (Frese and Keith, 2015). In turn, employees build mutual trust, receive interpersonal support, and satisfy their work and relational resource needs, reinforcing a secure organizational environment (Agarwal and Farndale, 2017). This psychological security motivates employees to allocate resources toward innovative problem solving rather than simply protecting existing resources (Hsu and Chen, 2017). Because bootleg innovation often entails violating organizational norms or circumventing restrictive regulations, employees with higher psychological safety—who feel fewer threats from the organization—are more inclined to undertake such behaviors (Qi et al., 2022; Chen et al., 2022).

Therefore, when an organization’s climate is tolerant of errors and actively encourages innovation, EMC strengthens psychological safety. Employees with greater psychological safety are, in turn, more likely to engage in bootleg innovation (Zhao et al., 2023). Based on these arguments, we propose:

*H2:* Psychological safety mediates the relationship between error management climate and bootleg innovation among new-generation employees.

**Figure 1:**
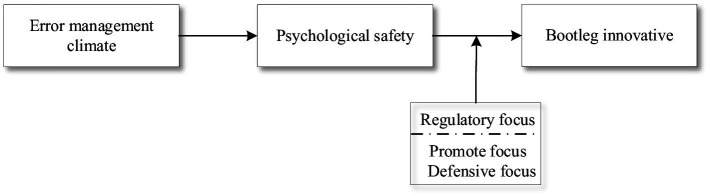
Illustrates the proposed theoretical model.

The research model is shown in Figure 1.

### 2.3 Moderating role of regulatory focus

Employees differ in their motivations toward risk and reward, which in turn shape their innovation behaviors (Gao et al., 2020). For new-generation employees, psychological safety supplies essential cognitive resources for engaging in bootleg innovation by alleviating fears of negative outcomes. Meanwhile, their self-regulatory orientation, whether focused on pursuing gains or avoiding losses, determines how they respond to psychological safety (Tenzer and Yang, 2019).

Employees with a strong promotion focus are driven by aspirations and potential gains, which makes them more willing to embrace uncertainty and take risks. They tend to challenge the status quo, pursue novel ideas, and adapt quickly to supportive organizational environments (Frese and Keith, 2015). When these younger employees experience high psychological safety, they feel empowered to follow their genuine ideas, question organizational rules, and engage in bootleg innovation. Even if their psychological safety is relatively low, promotion-focused individuals remain motivated by organizational goals and a sense of social responsibility, prompting them to take risks for the benefit of organizational development rather than strictly adhere to established norms.

In contrast, employees with a strong defensive focus prioritize security and risk avoidance. Even under conditions of high psychological safety, these individuals tend to be more cautious, sensitive to negative feedback, and committed to routine tasks. Their heightened concern about failure and punishment leads them to rely heavily on formal organizational guidelines and to emphasize error-free performance (Salancik and Pfeffer, 1978; Crick and Dodge, 1994). As a result, defensive-focused employees are less likely to explore unconventional innovation and may respond defensively when facing uncertainty. According to regulatory focus theory (Higgins et al., 1997; Lanaj et al., 2012), individuals possess two distinct self-regulatory systems: promotion and defensive, which help explain variations in behavioral styles (Gao et al., 2020). A sense of psychological safety provides cognitive resources that foster employees’ innovative behavior by alleviating concerns about engaging in bootleg innovative activities. However, it is also crucial to consider employees’ motivations for pursuing such behaviors. Employees with a high promotion focus emphasize potential gains and seek achievement, which aligns with traits associated with bootleg innovation, such as flexibility, willingness to challenge the status quo, and rapid adaptation to organizational climate (Augsdorfer, 2012). When their level of psychological safety is high, they are more likely to follow their genuine ideas, question organizational rules, and engage in bootleg innovation. Moreover, promotion-focused employees tend to exhibit a strong sense of responsibility and social identity, along with a transformational approach to innovation focused on organizational goals and interests. Even when their psychological safety is lower, these individuals are still willing to take risks to challenge the status quo and engage in prosocial behavior, which in this context can be considered bootleg innovation for organizational benefit (Jiang et al., 2023). Therefore, the positive effect of psychological safety on bootleg innovation behavior may be amplified for employees with a stronger promotion focus.

By contrast, employees with a strong defensive focus are more conservative, sensitive to negative feedback, and risk averse, even when experiencing high psychological safety (Crick and Dodge, 1994). Their self-centered focus and heightened sensitivity to potential failure lead them to rely heavily on explicit organizational information, prioritize error-free task completion, and emphasize adherence to rules. Consequently, defensive-focused employees are less likely to pursue non-normative innovation and may adopt defensive psychological responses when facing uncertainty (Salancik and Pfeffer, 1978; Crick and Dodge, 1994). Based on these insights, we propose the following hypotheses:

*H3:* Promotion focus positively moderates the impact of psychological safety on bootleg innovation among new-generation employees. The stronger an employee’s promotion focus, the stronger this relationship.

*H4:* Defensive focus negatively moderates the impact of psychological safety on bootleg innovation among new-generation employees. The stronger an employee’s defensive focus, the weaker this relationship.

Research indicates that individual traits shape how employees interpret information from their organizational environment during behavioral adjustment (Ning and Jin, 2009). In particular, when employees perceive that an error management climate enhances their psychological safety, those with a strong promotion focus are more likely to attend to and recall information about positive outcomes. Consequently, they feel compelled to take action aimed at achieving goals, advancing their careers, and seizing new opportunities (Lockwood et al., 2002). Thus, promotion-focused employees are inclined to view the open, inclusive, and supportive environment fostered by an error management climate as a way to manage future risks. They are generally more willing to embrace responsibility, take calculated risks, and engage in bootleg innovation. This tendency further amplifies the role of error management climate in stimulating bootleg innovation among new-generation employees by bolstering their psychological safety.

In contrast, social information processing theory suggests that defensive-focused employees, because of their cautious and risk-averse nature, are more sensitive to negative cues such as potential losses, punishments, and failures (Higgins, 1998; Ning and Jin, 2009). These individuals process information carefully to avoid tasks that might yield negative outcomes. Therefore, even when defensive-focused employees recognize that an error management climate enhances their psychological safety, they may choose to “wait for things to change” rather than proactively engage in bootleg innovation. This reluctance stems from a fear of failure and punishment, as well as a belief that such behavior is outside their role and could jeopardize existing resources. Based on these observations, we propose the following hypotheses:

*H5:* A promotion focus positively moderates the indirect effect of error management climate on employees’ bootleg innovation via psychological safety, such that the mediating effect is stronger under higher levels of promotion focus.

*H6:* A defensive focus negatively moderates the indirect effect of error management climate on employees’ bootleg innovation via psychological safety, such that the mediating effect is weaker under higher levels of defensive focus.

## 3 Study 1-method

### 3.1 Data collection and participants

This study was conducted between July and September 2024, focusing on new-generation employees in various enterprises. To minimize common method bias and capture causal relationships more effectively. Data were collected from employees in 23 organizations across five cities: Beijing, Shanghai, Shijiazhuang, Urumqi, and Kunming, at three distinct time points. Participants were selected through purposive sampling, ensuring a diverse representation of industries and firm sizes. To control for potential industry or firm size effects, we included these variables as control variables in our analysis (e.g., industry type and job level). The initial survey was administered in July 2024, followed by two additional waves at approximately two-week intervals.

To ensure data authenticity and quality, the survey included reverse-coded items (e.g., “My job plan requires me to focus all my energy on the organization’s assigned tasks, leaving no time for other work”) and attention-check questions (e.g., Please select ‘very satisfied’ or ‘very dissatisfied’). Data were collected using a combination of online and offline methods for accessibility. To manage potential biases between online and offline survey modes, we ensured that the survey design and content were identical across both methods. Additionally, we implemented data matching procedures using the last four digits of participants’ mobile phone numbers, allowing us to verify consistency across waves and account for potential discrepancies between the two modes. This approach helped to minimize mode effects and enhance the reliability of the data.

The first wave measured demographic variables, error management climate, promotion focus, and defensive focus; the second wave measured psychological safety; the third wave assessed bootleg innovation. To ensure data validity, responses with missing key variables, logically inconsistent answers, or less than one year of work experience were excluded. The latter criterion was applied to remove individuals likely still undergoing initial onboarding and training without substantive job tasks. After rigorous screening and matching, 387 valid responses were retained, yielding an effective response rate of approximately 87.95%.

The final sample was demographically diverse. In terms of gender, 54.8% of respondents were male and 45.2% female. The largest age group was 26–30 years (43.9%), followed by 20–25 years (34.6%) and 31–35 years (21.5%). Regarding educational background, 70.0% held a bachelor’s degree, 26.6% had a master’s degree or higher, 2.6% had a junior college diploma, and 0.8% had completed high school or less. With respect to job roles, 58.1% were frontline employees, 22.7% were first-line managers, 14.5% held middle management positions, and 4.7% were senior managers. The sample spanned a variety of industries: 26.4% worked in IT, 25.8% in information technology services, 17.3% in manufacturing, 15.8% in internet finance, and 14.7% in high-tech sectors.

### 3.2 Measurement scales

The scales used in this study are well-established and widely recognized in both domestic and international authoritative literature. A rigorous translation and back-translation procedure was followed to adapt these scales, ensuring the reliability and validity of the questionnaire within the Chinese context. Prior to the formal data collection, a pilot study was conducted with a small sample, and the questionnaire was revised based on the feedback received. The scales employed a 5-point Likert scale, with scores ranging from 1–5 indicating the respondents’ level of agreement with each item.

#### 3.2.1 Error management climate

Based on the EMC theory scale and following Van’s approach, a positively framed error management climate scale was selected for this study (Van Dyck et al., 2005). Although this scale was originally designed to measure Error Management Culture, which focuses on the underlying organizational norms and values, its items emphasize observable practices that employees can perceive and experience. This makes the scale highly suitable for assessing Error Management Climate, defined as the shared perceptions of organizational practices. As such, the scale’s focus on visible practices such as how errors are discussed, corrected, and managed aligns with the definition of climate, which emphasizes employees’ perceptions of their immediate work environment (Marquardt et al., 2012, 2021; Schein, 1992). This scale consists of 16 items, with example items such as “When an error occurs, it is corrected immediately” and “I can ask others for advice when I make a mistake.” Numerous domestic studies have validated the reliability and validity of this scale, with the present study reporting a Cronbach’s *α* coefficient of 0.919.

#### 3.2.2 Psychological safety

This scale is based on a 5-item measure developed by Zhang and Wang (2019). A sample item is “I can express myself freely.” In this study, the scale had a Cronbach’s *α* coefficient of 0.921.

#### 3.2.3 Bootleg innovation

This scale is based on a 5-item measure developed by Criscuolo et al. (2014). An example item is “I enjoy thinking about new ideas beyond my main job responsibilities.” In this study, the Cronbach’s α coefficient for this scale was 0.882.

#### 3.2.4 Regulatory focus

The scale is based on an 8-item measure developed by Lockwood et al. (2002), with 4 items for a promotion focus (PF) and 4 items for a defensive focus (DF). Sample items include “I think a lot about how I can be successful” and “In general, I am more concerned about how I can avoid failure than how I can achieve success.” The Cronbach’s alpha coefficients for the promotion focus and defensive focus scales in this study were 0.890 and 0.808, respectively.

#### 3.2.5 Control variables

As recommended by previous studies (Gao et al., 2020), we included gender, age, education, working time, job level, and industry type as control variables. Table 1 presents the demographic characteristics of the respondents.

**Table 1:** Descriptive statistics of the main variables.

Control variable	1	2	3	4	5	6	7	8	9	10
1. Gender	1									
2. Age	−0.075	1								
3. Education	−0.033	−0.023	1							
4. Job level	−0.034	−0.045	−0.106^*^	1						
5. Industry type	−0.154^**^	0.258^**^	−0.074	0.048	1					
6. Error management climate	−0.085	0.185^**^	0.006	0.029	0.055	1				
7. Psychological safety	−0.201^**^	0.178^**^	−0.060	0.012	0.082	0.238^**^	1			
8. Bootleg innovation	−0.201^**^	0.186^**^	−0.076	−0.027	0.124^*^	0.263^**^	0.655^**^	1		
9. Promotion focus	0.317^**^	−0.110^*^	0.049	0.049	−0.102^*^	−0.031	−0.219^**^	−0.254^**^	1	
10. Defensive focus	−0.195^**^	0.083	−0.037	−0.094	0.123^*^	0.174^**^	0.455^**^	0.542^**^	−0.216^**^	1
Mean	3.188	1.694	2.365	2.063	2.425	3.297	3.024	3.113	2.658	3.092
SD	1.074	0.461	0.925	0.785	0.724	1.074	0.563	0.441	0.845	0.531

*p < 0.05, **p < 0.01; ***p < 0.001.

### 3.3 Reliability and validity

The Cronbach’s alpha values for an error management climate, psychological safety, bootleg innovation, promotion regulatory focus, and defensive regulatory focus were 0.919, 0.921, 0.882, 0.890, and 0.808, respectively. All of these values were above 0.7, indicating good reliability of the questionnaire. The average variance extracted (AVE) values for each variable were 0.522, 0.761, 0.680, 0.753, and 0.636, all exceeding the 0.5 threshold, suggesting good convergent validity. Furthermore, the composite reliability (CR) values were 0.946, 0.941, 0.914, 0.924, and 0.875, which were all greater than the recommended value of 0.7, further confirming the strong convergent validity of the data. Discriminant validity is assessed in Table 2, where the indicators of the five-factor model (χ^2^/df = 2.095, SRM*R* = 0.0496, RMSEA = 0.053, IFI = 0.920, TLI = 0.912, CFI = 0.919) exceed the critical values. The model’s goodness of fit is superior to that of the other models, indicating strong discriminant validity among the variables.

**Table 2:** Results of the confirmatory factor analysis.

Measurement model	χ^2^/df	CFI	TLI	IFI	RMSEA	SRMR
Five-factor model (EMC, PS, BI, PF, DF)	2.095	0.919	0.912	0.920	0.053	0.0496
Four-factor model (EMC, PS, BI, PF + DF)	3.147	0.840	0.828	0.841	0.075	0.1026
Three-factor model (EMC, PS, BI+PF + DF)	3.992	0.776	0.760	0.777	0.088	0.0816
Two-factor model (EMC, PS + BI+PF + DF)	4.807	0.714	0.695	0.715	0.099	0.0883
Single-factor model (EMC + PS + BI+PF + DF)	8.682	0.421	0.384	0.424	0.141	0.1716

*N* = 387; EMC refers to the error management climate, PS refers to psychological safety, BI refers to bootleg innovation, PF represents a promotion focus, and DF represents a defensive focus. “+” means that two factors are merged into one.

### 3.4 Common method variance test

Although a three-phase strategy was employed, common method bias could still affect the self-reported data on error management climate (EMC), psychological safety (PS), bootleg innovation (IB), promotion focus (PF), and defensive focus (DF). To address this potential bias, we performed Harman’s single-factor test by conducting an exploratory factor analysis (EFA) on the unrotated factor solution, which included 16 items for EMC, 5 items for PS, 5 items for IB, and 8 items for RF. The results of the EFA indicated that no single factor accounted for the majority of the covariance in the data. Specifically, 10 factors explained 65.4% of the total variance, with the largest single factor accounting for 27.26%. Thus, common method bias was not a significant concern.

### 3.5 Study 1- results

#### 3.5.1 Descriptive statistics

Descriptive statistics for all variables are presented in Table 1. As shown, a significant positive correlation was found between error management climate and bootleg innovation (*r* = 0.263, *p* < 0.01). Additionally, positive correlations were observed between an error management climate and psychological safety (*r* = 0.238, *p* < 0.01) and between psychological safety and bootleg innovation (*r* = 0.655, *p* < 0.01), all of which were statistically significant. These results aligned with theoretical expectations and supported the subsequent hypothesis testing (Figure 1).

#### 3.5.2 Hypothesis tests

This study selected gender, age, education, years of work, position, and industry type as control variables. The regression results for direct, mediating, and moderating effects obtained via hierarchical regression analysis are presented in Table 3.

**Table 3:** Results of the hierarchical regressions.

Variable	Psychological safety		Bootleg innovation					
	M_1_	M_2_	M_3_	M_4_	M_5_	M_6_	M7	M8
Gender	−0.195***	−0.178***	−0.192***	−0.174***	−0.071	−0.066	−0.045	−0.041
Age	0.101	0.099	0.073	0.070	0.010	0.011	0.017	0.011
Education	−0.033	−0.051	−0.040	−0.060	−0.019	−0.029	−0.037	−0.010
Job level	−0.016	−0.011	−0.067	−0.061	−0.057	−0.055	−0.052	−0.026
Industry type	0.001	0.005	0.044	0.048	0.044	0.045	0.047	0.018
Error management climate		0.196***		0.219***		0.101*	0.571***	0.492***
Psychological safety					0.623***	0.603***	−0.117**	
Promotion focus								0.301***
Defensive focus							0.143***	
Psychological safety×promotion focus								−0.108**
Psychological safety×defensive focus							0.471	0.526
R^2^	0.074	0.110	0.086	0.131	0.445	0.454	0.018	0.011
△R^2^	0.074	0.036	0.086	0.044	0.359	0.324	37.255***	46.451***
F	5.097***	6.702***	5.971***	8.134***	43.463***	39.340***	M9	M11

*N* = 387; *p < 0.05; **p < 0.01; ***p < 0.001.

The regression results in Table 3 indicate that an error management climate positively influences the bootleg innovation of new-generation employees (M4: *β* = 0.219, *p* < 0.001), the R^2^ value for Model 4 is 0.131, and it increased by 0.045 from Model 3 (R^2^ = 0.086), confirming Hypothesis 1. The mediating role of psychological safety in the relationship between an error management climate and bootleg innovation was further tested. As shown in the M2 and M5 models, an error management climate has a positive effect on psychological safety (*β* = 0.196, *p* < 0.001), and psychological safety significantly impacts the bootleg innovation of new-generation employees (*β* = 0.623, *p* < 0.001). Thus, Hypothesis H2 was preliminarily supported. When psychological safety was introduced into the M6 model with bootleg innovation as the dependent variable, the coefficient for the effect of an error management climate decreased (*β* = 0.101, *p* < 0.05), whereas the effect of psychological safety remained significant (*β* = 0.603, *p* < 0.001), the R^2^ value for Model 5 is 0.454, which increased by 0.009 from Model 4 (R^2^ = 0.445). These findings suggest that psychological safety plays an important mediating role in the relationship between an error management climate and bootleg innovation. Therefore, Hypothesis H2 was further supported. Additionally, when the PROCESS plug-in in SPSS 27.0 was used with bootstrapping to verify Hypothesis H2, the indirect effect of an error management climate on bootleg innovation was 0.1182, with a confidence interval of [0.0480, 0.1947]. Since the confidence interval did not contain zero, the mediating effect was statistically significant, providing further support for Hypothesis H2.

Before conducting regression analysis, this study standardized both the independent variable and moderator separately to address multiple social concerns and subsequently constructed the interaction terms (Edwards and Lambert, 2007). The specific analysis results are presented in Table 3 for Models 7 and 8. The interaction between employees’ psychological safety and promotion focus positively affects the bootleg innovation of new-generation employees (*β* = 0.143, *p* < 0.001), supporting Hypothesis H3. In contrast, the interaction between employees’ psychological safety and bootleg focus negatively impacts the bootleg innovation of new-generation employees (*β* = −0.108, *p* < 0.01), supporting Hypothesis H4.

**Figure 2:**
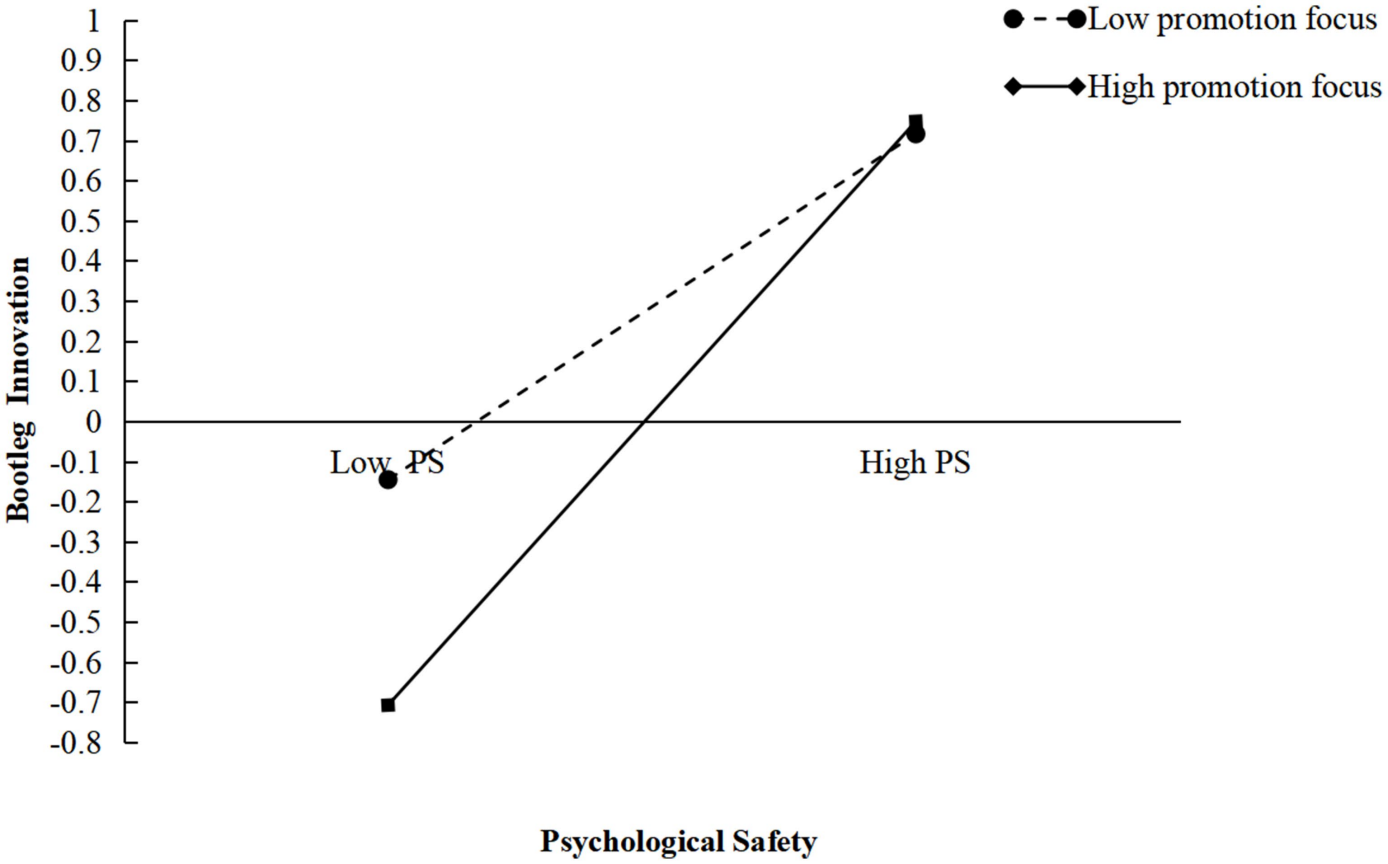
Interaction effect between psychological safety and promotion focus on bootleg innovation, Study 1.

**Figure 3:**
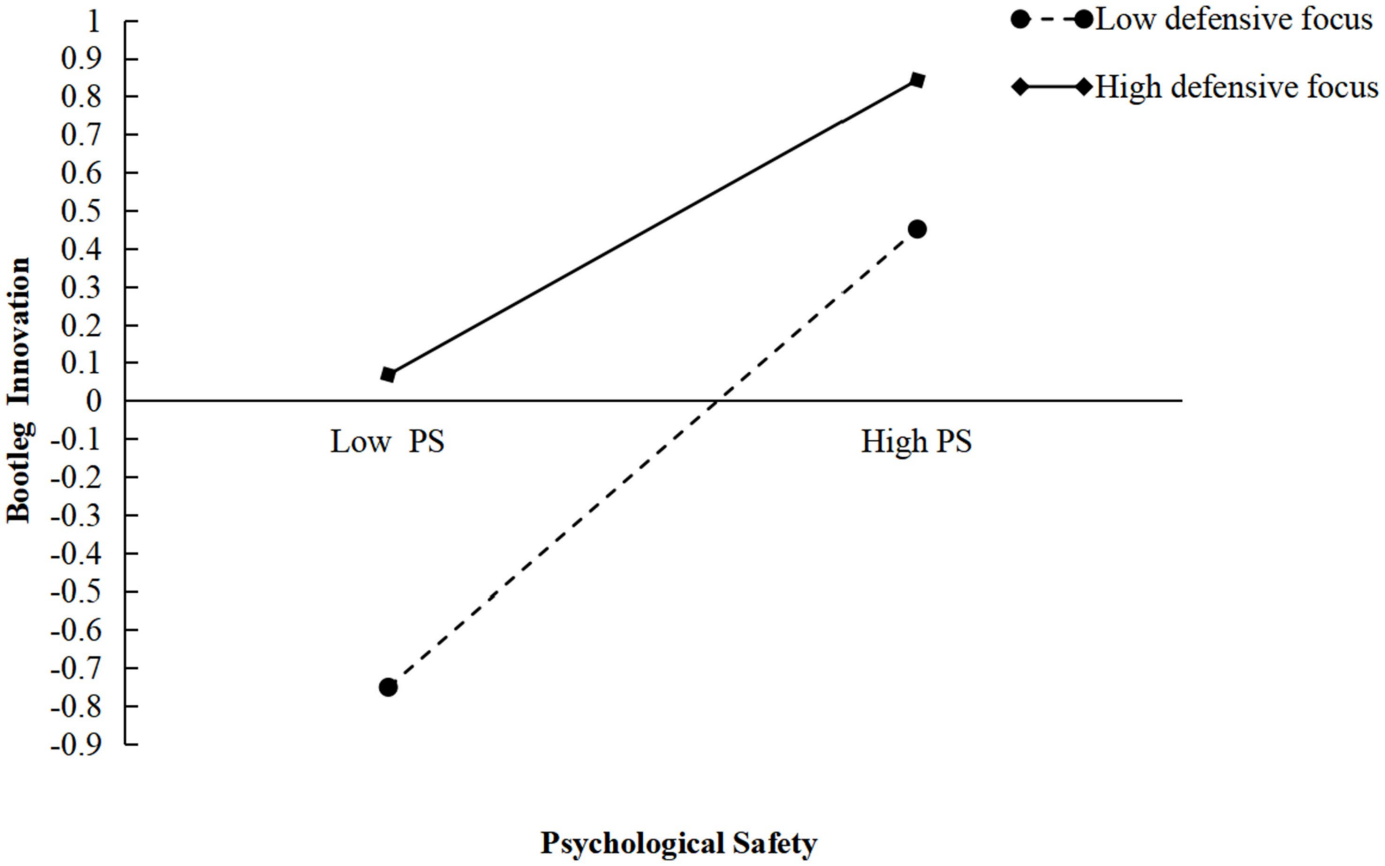
Interaction effect between psychological safety and defensive focus on bootleg innovation, Study 1.

To further clarify the direction and magnitude of the moderating effects of focus, this study employed the method proposed by Aikeni and West, 1991 to generate moderation plots, as illustrated in Figures 2, 3. As shown in Figure 2, the slope corresponding to a high promotion focus is steeper than that corresponding to a low promotion focus, indicating a positive moderating effect of a promotion focus on the relationship between psychological safety and bootleg innovation among new-generation employees. Similarly, Figure 3 shows that the slope associated with a high defensive focus is less steep than that associated with a low defensive focus, suggesting a negative moderating effect of a defensive focus on the relationship between psychological safety and bootleg innovation. These results provided further support for H3 and H4.

Additionally, the SPSS 27.0 process plugin was used to validate Hypotheses H3 and H4 with Model 1 and Bootstrap, as shown in Table 4. Specifically, when the moderation variable, promotion focus, was set to a low value, the effect of psychological safety on bootleg innovation was 0.4276, with a lower confidence interval (LCI) of [0.3013, 0.5540], which does not include 0, indicating a significant moderating effect of a promotion focus. When promotion focus was set to a high value, the effect of psychological safety on bootleg innovation increased to 0.7145, with an LCI of [0.6179, 0.8111], also excluding 0, further supporting the significant moderating effect of a promotion focus. Moreover, as the level of promotion focus increased from low to high, the positive effect of psychological safety on bootleg innovation strengthened (from *β* = 0.4276 to *β* = 0.7145), which provided additional evidence for Hypothesis H3. Similarly, when the moderation variable, defensive focus, was set to a low value, the effect of psychological safety on bootleg innovation was 0.5970, with an LCI of [0.4914, 0.7027], excluding 0, indicating a significant moderating effect of a defensive focus. When defensive focus was set to a high value, the effect decreased to 0.3785, with an LCI of [0.2690, 0.4881], again excluding 0, confirming a significant moderating effect of a defensive focus. However, as defensive focus decreased from high to low, the negative effect of psychological safety on bootleg innovation weakened (from *β* = 0.5970 to *β* = 0.3785), providing further support for Hypothesis H4.

**Table 4:** Results of the moderating effect test for moderating focus.

Moderator variable	Effect	BootSE	BootLLCI	BootULCI
Low promotion focus	0.4276	0.0643	0.3013	0.5540
Medium promotion focus	0.5711	0.0408	0.4908	0.6513
High promotion focus	0.7145	0.0491	0.6179	0.8111
Low defensive focus	0.5970	0.0537	0.4914	0.7027
Medium defensive focus	0.4878	0.0409	0.4074	0.5682
High defensive focus	0.3785	0.0557	0.2690	0.4881

Following Edwards’ approach, this study employed the bootstrap method with 5,000 resamples to test Hypotheses H5 and H6 at the 95% confidence level. The sample data were divided into high and low groups based on the mean plus or minus one standard deviation of the moderating variable to examine the differences in the “conditional indirect effect” of an error management climate on bootleg innovation among new-generation employees under varying levels of regulatory focus. When the promotion focus was low, the indirect effect of the error management climate on bootleg innovation was 0.0823, with a lower confidence interval (LCI) of [0.0316, 0.1448], excluding 0. When the promotion focus was high, the effect increased to 0.1350, with an LCI of [0.0569, 0.2200], again excluding 0. The difference in effects between the two levels of promotion focus was 0.0527, with an LCI of [0.0143, 0.1081], excluding 0. Therefore, Hypothesis H5 was supported. Similarly, when the defensive focus was low, the indirect effect of the error management climate on bootleg innovation was 0.1135, with an LCI of [−0.0448, −0.1914], excluding 0. When the defensive focus was high, the effect decreased to 0.0739, with an LCI of [−0.0292, −0.1308], excluding 0. The difference in effects between the two levels of defensive focus was −0.0396, with an LCI of [−0.0885, −0.0016], excluding 0. Therefore, Hypothesis H6 was also supported.

## 4 Study 2-method

Study 2 was conducted as an exploratory study, with a scenario-based experiment involving 200 participants. This study was designed to investigate the potential effects of error management climate on bootleg innovation and did not follow a preregistration process.

### 4.1 Procedure and experiment design

Following previous research studies (He et al., 2025; Zhang et al., 2025), we recruited a sample of 200 new-generation employees via the Credamo platform. All responses were anonymized to protect data privacy. The final sample consisted of 69.0% male (*n* = 138), with an average age of 25.6 years (SD = 5.7), and 79.0% reporting over one year of work experience.

### 4.2 Manipulation of error management climate

The manipulation of error management climate in this study was based on the conceptual framework and scale items developed by Van Dyck et al. (2005). Participants were randomly assigned to one of two experimental conditions: a high error management climate condition or a low error management climate condition. To ensure that the manipulation was clear and relatable, both conditions were framed within the context of a new employee’s early experiences in a workplace environment. Specifically, both conditions presented a scenario where:

“A new employee, during their first three months at the company, was tasked with participating in the development of a new product. Throughout the process, they proposed several unprecedented design ideas, some of which were highly innovative but not yet fully developed.”

Although the basic scenario remained the same across both conditions, the crucial difference lay in how the new employee’s contributions and errors were treated by the team and management, reflecting the differing levels of error management climate.

#### 4.2.1 High error management climate

In this condition, the new employee’s innovation ideas were embraced by the team, and management provided constructive feedback, framing mistakes as learning opportunities. The atmosphere was one of psychological safety, where errors were openly discussed, and employees felt supported in their attempts to innovate, even if their ideas were not fully formed.

#### 4.2.2 Low error management climate

In contrast, the low error management culture condition described a scenario where the new employee’s innovation ideas were met with skepticism and criticism. Mistakes were viewed negatively, and management focused more on the flaws in the employee’s ideas rather than on opportunities for growth. Feedback was more critical, and the overall tone of the work environment was one of low psychological safety, where errors were penalized and viewed as a sign of incompetence.

After reading their assigned condition instructions, participants first provided demographic information and then engaged with the condition materials. Finally, they completed a questionnaire assessing the perceived level of error management climate, bootleg innovation, psychological safety.

### 4.3 Measures

In the survey 2, participants completed the same scales used in Study 1 to measure error management climate (*α* = 0.866), bootleg innovation (*α* = 0.809), and psychological safety (*α* = 0.779).

### 4.4 Results

#### 4.4.1 Manipulation check

**Figure 4:**
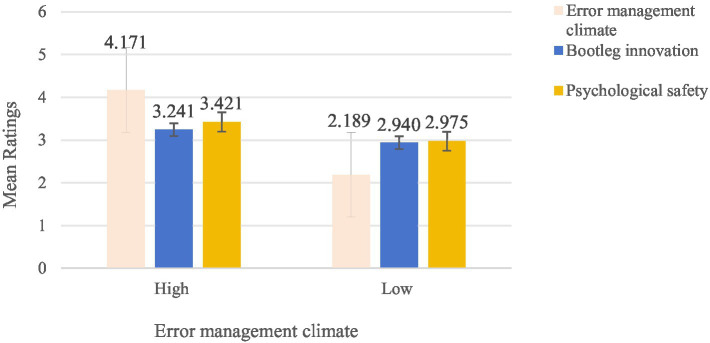
Perceptions of error management climate, bootleg innovation, psychological safety. Error bars represent ± 1 standard error.

To verify the effectiveness of our experimental manipulations, we conducted an Independent Samples t-test with the experimental condition (1 = high error management climate; 2 = low error management climate) as the independent variable and the manipulation check scores as the dependent variable. Participants in the high error management climate condition rated significantly higher levels of error management climate (*M* = 4.171, SD = 0.521) compared to those in the low error management climate condition (*M* = 2.189, SD = 0.465), t (198) = −24.562, *p* < 0.001, *d* = −1.562, supporting the effectiveness of the manipulation. Figure 4 depicts the key results.

#### 4.4.2 Hypothesis tests

As in the pilot study, we conducted an independent samples *t*-test with experimental condition (1 = high error management climate; 2 = low error management climate) as the independent variable and bootleg innovation as the dependent variable to test Hypothesis 1. Results showed that participants reported significantly higher bootleg innovation under the high error management climate condition (*M* = 3.241, SD = 0.386) than under the low error management climate condition (*M* = 2.940, SD = 0.326), *t* (198) = −11.79, *p* < 0.001, *d* = −1.67. An OLS regression further confirmed a significant positive relationship between error management climate and turnover intention (*b* = 0.159, *p* < 0.001). Thus, Hypothesis 1 was supported, suggesting that a positive error management climate encourages bootleg innovation. Additionally, participants reported significantly higher psychological safety under the high error management climate condition (*M* = 3.421, SD = 0.353) compared to the low error management climate condition (*M* = 2.975, SD = 0.392), t (198) = −7.326, *p* < 0.001, *d* = −1.67. An OLS regression confirmed a significant positive relationship between error management climate and psychological safety (*b* = 0.446, *p* < 0.001).

To test whether psychological safety mediates the relationship between error management climate and bootleg innovation, we incorporated the dichotomous variable for error management climate (1 = high error management climate; 2 = low error management climate) into a regression model. Using PROCESS Model 4, we performed a bootstrapping path analysis with 5,000 random samples from the full dataset. The results are summarized in Table 5.

**Table 5:** Results of the hierarchical regressions.

Variable	Psychological safety		Bootleg innovation	
	*b*	SE	*b*	SE
Gender	2.975***	0.043	1.998***	0.226
Error management climate	0.446***	0.061	0.159*	0.065
Psychological safety			0.317***	0.075
*R* ^2^	0.266		0.244	

The analysis revealed a significant positive effect of error management climate on psychological safety (*b* = 0.446, SE = 0.061, *p* < 0.001), and a significant positive effect of psychological safety on bootleg innovation (*b* = 0.317, SE = 0.075, *p* < 0.001). The indirect effect was 0.141, with a standard error of 0.038, and a 95% confidence interval of [0.074, 0.223], which does not include zero. This indicates that psychological safety significantly mediates the relationship between error management climate and bootleg innovation, thereby supporting Hypothesis 3.

## 5 Conclusion and discussions

### 5.1 Research findings

This paper applies resource conservation theory and social information processing theory to examine the stimulation mechanism through which employees’ perceived organizational error management climate influences the bootleg innovation of the new generation of employees. Specifically, this study explores this relationship from the perspective of errors and tests the mediating role of psychological safety. Additionally, it investigates the moderating effect of characteristic focus. The results show that (1) an error management climate positively affects bootleg innovation among new-generation employees; (2) psychological safety mediates the relationship between error management climate and bootleg innovation; and (3) a promotion focus positively moderates the mediating effect of error management climate on bootleg innovation, whereas a defensive focus negatively moderates this effect.

### 5.2 Theoretical contributions

This paper applies Conservation of Resources (COR) theory to explore how error management climate (EMC) influences bootleg innovation, advancing our understanding of how organizational climates shape individual behaviors. Previous research on bootleg innovation has predominantly concentrated on leadership styles, while the role of organizational climate (Wu et al., 2022), particularly error management climate, remains underexplored. By focusing on EMC, this study offers a novel theoretical perspective that integrates the broader organizational climate with individual-level innovation behaviors. This shift in focus not only fills a gap in the existing literature but also broadens the theoretical framework for understanding how environmental factors, such as tolerance for mistakes, drive employee innovation, particularly in the context of new-generation employees (Du and Cao, 2014).

Second, previous research has not deeply explored the mechanisms underlying an error management climate. Building on the conservation of resources theory, this study introduces psychological safety as a mediator in the relationship between this climate and bootleg innovation among new-generation employees. It systematically constructs and tests a model that examines the interactions among an error management climate, psychological safety, and bootleg innovation by these employees. This approach not only uncovers the intrinsic mechanisms through which an error management climate influences bootleg innovation but also validates the mediating role of psychological safety in this process. The findings are consistent with those of Zhang et al. (2024) and others, suggesting that a positive organizational climate fosters employees’ psychological safety, thereby encouraging them to break organizational norms in pursuit of new breakthroughs. Consequently, this study contributes to the literature on the antecedents of bootleg innovation, offering a novel perspective on the mechanisms by which an error management climate exerts its influence.

Finally, social information processing theory highlights the positive role of trait regulatory focus in promoting bootleg innovation. Employees with a highly promotion-focused regulatory orientation are more inclined to embrace environmental changes, adapt to shifting conditions, and adjust their actions in alignment with their goals. In contrast, employees with a high defensive-focused regulatory orientation tend to adhere to established norms and adopt a more cautious, “wait-and-see” approach when faced with environmental changes. On this basis, the current study suggests that when employees experience high levels of psychological safety—fostered by an error management atmosphere that emphasizes forgiveness—those with a promotion-focused regulatory orientation are more likely to deviate from organizational norms and engage in innovation that breaks tradition. Furthermore, as a positive personality trait, a promotion-focused regulatory orientation, when combined with inclusive information from an error management atmosphere that fosters psychological safety, encourages employees to adopt constructive approaches in response to organizational change.

### 5.3 Practice implications

The conclusions drawn from this study carry significant practical implications for management. Enterprises should acknowledge that errors are an inherent part of the innovation process and adopt a rational approach towards mistakes made by employees during innovation activities. It is crucial to navigate organizational norms with flexibility, respect the diverse innovation approaches employed by different employees, and mitigate conflicts arising between employees’ bootleg innovation and organizational standards. Specific recommendations are outlined below: Organizations should foster an error management climate that emphasizes tolerance for mistakes, as this can enhance psychological safety and encourage bootleg innovation. However, the findings also carry global implications. In the context of multinational organizations and digital-based companies, the application of error management climate is especially crucial. In digital environments where employees work autonomously and remotely, creating an error-tolerant culture can help mitigate the risks of failure and encourage innovation. Furthermore, in multinational organizations with diverse cultural backgrounds, it is important for managers to understand how cultural differences in attitudes towards failure or hierarchical values can influence employees’ willingness to engage in bootleg innovation. This study provides valuable insights for how organizations across sectors and regions can strategically cultivate a climate for innovation, thereby enhancing both creativity and organizational performance.

### 5.4 Limitations and perspectives

While this study provides valuable insights into the role of error management climate in bootleg innovation, there are several limitations that future research could address. First, cultural context plays a critical role in shaping employees’ perceptions of error management climate and psychological safety. Given that our data were collected in China, it is important to consider how cultural factors such as collectivism or hierarchical values may influence the findings. Future research could explore bootleg innovation in different cultural settings to assess whether these findings hold true across diverse populations (Hirschi et al., 2025). Second, our study did not separately analyze the dimensions of error management climate (e.g., tolerance for mistakes, error communication). Future studies could investigate these dimensions in greater detail to understand their individual impacts on innovation behavior (Abbasi et al., 2024). Finally, while this study focuses on new-generation employees, future research could expand the sample to include employees from different age groups or industries, providing a broader perspective and enhancing the generalizability of the findings.

## Data Availability

The original contributions presented in the study are included in the article/supplementary material, further inquiries can be directed to the corresponding author/s.
